# Clinical trial on the efficacy of exhaled carbon monoxide measurement in smoking cessation in primary health care

**DOI:** 10.1186/1471-2458-12-322

**Published:** 2012-07-04

**Authors:** Joana Ripoll, Helena Girauta, Maria Ramos, David Medina-Bombardó, Agnès Pastor, Cristina Alvarez-Ossorio, Lucía Gorreto, Maria Esteva, Elena García, Ana Uréndez, Ana Buades, Elena Torres

**Affiliations:** 1 Primary Care Research Unit of Mallorca, Baleares Health services-IbSalut, Mallorca, Spain; 2 Public Health Department, Balearic Islands Health Department, Mallorca, Spain; 3 Manacor Health Care Centre, Baleares Health services-IbSalut, Mallorca, Spain; 4 Sineu Health Care Centre, Baleares Health services-IbSalut, Mallorca, Spain; 5 Camp Redó Health Care Centre, Baleares Health services-IbSalut, Mallorca, Spain; 6 Emili Darder Health Care Centre, Baleares Health services-IbSalut, Mallorca, Spain; 7 Artà Health Care Centre, Baleares Health services-IbSalut, Mallorca, Spain; 8 Sant Agustí Health Care Centre, Baleares Health services-IbSalut, Mallorca, Spain; 9 Son Pisà Health Care Centre, Baleares Health services-IbSalut, Mallorca, Spain

## Abstract

**Background:**

Smoking cessation is beneficial for our health at any point in life, both in healthy people and in people already suffering from a smoking-related disease. Any help to quit smoking can produce considerable benefits for Public Health. The purpose of the present study is to evaluate the efficacy of the CO-oximetry technique together with brief advice in smoking cessation, in terms of reduction of the number of cigarettes or in the variation of the motivation to quit smoking at month 12 compared with brief advice alone.

**Methods/Design:**

Randomised, parallel, single-blind clinical trial in a primary health care setting in Majorca (Spain). Smokers in contemplation or pre-contemplation phase will be included in the study. Exclusion criteria: Smokers in preparation phase, subjects with a terminal illness or whose health status does not allow them to understand the study or complete the informed consent, and pregnant or breastfeeding women. The subjects will be randomly assigned to the control group (CG) or the intervention group (IG). The CG will receive brief advice, and the IG will receive brief advice together with a measurement of exhaled CO. There will be follow-up evaluations at 6 and 12 months after inclusion. 471 subjects will be needed per group in order to detect a difference between groups ≥ 5%. Primary outcome: sustained smoking cessation (at 6 and 12 months) confirmed by urine cotinine test. Secondary outcomes: point smoking cessation at 6 and 12 months both confirmed by urine cotinine analysis and self-reported, reduction in cigarette consumption, and variation in phase of smoking cessation.

**Discussion:**

CO-oximetry is an inexpensive, non-invasive, fast technique that requires little technical training; making it a technique for risk assessment in smokers that can be easily applied in primary care and, if proven effective, could serve as a reinforcement aid in smoking cessation intervention activities.

**Trial Registration:**

Current Controlled Trials ISRCTN67499921

## Background

Tobacco smoking is the first cause of avoidable death in industrialised countries [1]. It has been known for many years that the negative effects of tobacco smoking on health are numerous [2,3]. Tobacco smoking is involved in the appearance of different types of cancer. It is also the main risk factor for cardiovascular disease, the most common known cause of chronic obstructive pulmonary disease, and many other health problems. Despite wide knowledge on the subject, the prevalence of tobacco smoking is still high. According to the 2006 National Health Survey, 31.56% of adult men and 21.51% of women are daily smokers, of whom 79% of men and 70% of women smoke more than 10 cigarettes per day.

Smoking cessation is beneficial for health at any point in life, both in healthy people and in people already suffering from a smoking-related disease [4]. However, smoking cessation is not an easy task, given that nicotine is a drug that generates great addiction [5].

According to article 12 of the Spanish Act 28/2005 (art. 12 Ley 28/2005) based on health measures for tobacco smoking and the regulation of its sale, supply, consumption and the advertising of tobacco products, the Spanish public Administrations are to promote the development of health programmes for smoking cessation in the Spanish Health System, especially in Primary Care. Likewise, National Health System strategies for cancer, ischemic cardiopathy and chronic obstructive pulmonary disease include reducing the prevalence of tobacco smoking amongst their objectives.

Although many smokers claim to have quit smoking on their own [6], others require specific help. Primary care doctors and nurses see most of the smokers at least once a year in their practice and have an excellent opportunity to diagnose tobacco smoking, evaluate the motivation behind it, and help them quit smoking. There is growing evidence on how healthcare professionals can help smokers end their addiction through pharmacological and non-pharmacological therapies [7]. The latter include: brief advice given by a doctor or a nurse, the delivery of self-help materials or more complex or intensive interventions based on motivational interviews and cognitive-behavioural techniques for psychological support, sometimes combined with pharmacological therapies [8].

The majority of non-pharmacological interventions for smoking cessation use the so-called transtheoretical model of change described by Prochaska and DiClemente at the beginning of the 90s [9]. According to this model, the smoker goes through a series of phases during his/her smoking cessation process: pre-contemplative (the smoker does not contemplate quitting smoking), contemplative (the smoker begins to be unhappy with his/her addiction and starts thinking about quitting smoking within the next 6 months), preparative (the smoker is prepared to quit smoking), smoking cessation and the eventual relapses that re-start the cycle.

The brief advice given by a healthcare professional can achieve between 1–3% smoking cessations after 6 months, taking into consideration that an additional 2–3% manage to quit smoking without help. Therefore, this measure is moderately effective, although it has a great impact across the population [10] and the percentage can be increased if the advice is not as brief and/or if it is accompanied by self-help and/or follow-up materials [11]. In order to increase the rates of smoking cessation by means of brief advice, another strategy could be the evaluation of the physical effects of smoking using physiological measures that offer the smoker some feedback on the effects of smoking, as happens with hypertensive patients and blood pressure readings [12].

These interventions are based on the hypothesis that one of the reasons why people continue smoking, in spite of knowing the harmful effects of tobacco, is that they underestimate the personal risk of becoming ill because of it. In this sense, the interventions will offer motivational feedback to promote awareness of the risk [13]. It has been suggested that some smokers who manage to quit smoking are more aware of the adverse effects of tobacco or to have had their health seriously compromised [14,15].

Three different types of feedback have been established: the first type analyses biomarkers of exposure to tobacco (nicotine, carbon monoxide); the second offers information regarding the risk of tobacco-related diseases (predisposition to lung cancer according to the CYP2D6 genotype); and the third describes the harmful effects of smoking (atherosclerotic plaques or worsening of pulmonary function) [15]. Isolated studies have provided mixed results regarding the effect of biomedical risk assessment as an aid for smoking cessation. A recent systematic review [16] concludes that, due to the lack of good quality evidence, it is not possible to draw firm conclusions. The following methodological recommendations are made: use adequate sample sizes, use allocation concealment procedures, agree on a definition of “abstinence” and systematically introduce measures to biochemically confirm it and, finally, carry out analyses by intention to treat. The measurement of CO levels in exhaled air by CO-oximetry is used to evaluate the degree of smoking by the smoker given that, in general, there is a direct relationship between the levels of CO and the number of cigarettes smoked [17]. The measurement of exhaled CO is also the preferred measurement to confirm tobacco abstinence [18].

It has been observed that the measurement of CO in exhaled air in smokers could be an indicative test of immediate and future harm to their health as a consequence of smoking [19] and this could increase their motivation to stop smoking, which could lead to smoking cessation in these patients. In the review mentioned above [16], three studies that measured the direct effect of CO-oximetry on smoking cessation [20-22] were identified. Two of them were carried out in primary care settings [20,21]. No positive changes in the rate of smoking cessation were observed.

The measurement of CO levels in exhaled air by CO-oximetry is an inexpensive, non-invasive, fast technique that requires little technical training [23] making it a technique for risk assessment in smokers that can be easily applied in primary care and, if proven effective, could serve as a reinforcement aid in smoking cessation intervention activities.

Therefore, we think it would be interesting to study its possible effect on smoking cessation to a greater extent through the design of a new study incorporating the methodological recommendations given by the experts, and by providing more robust results to determine efficacy.

The purpose of the present study is to evaluate the efficacy of the CO-oximetry technique, together with brief advice, in smoking cessation at month 12 in smokers in contemplative or pre-contemplative phase compared with brief advice alone. Furthermore, the study also aims to evaluate the efficacy of the CO-oximetry technique together with brief advice in the reduction of the number of cigarettes and in the variation of the motivation to quit smoking at month 12 in smokers in contemplative or pre-contemplative phase compared with brief advice alone.

## Methods/Design

### Design and settings

A randomised, parallel, single-blind clinical trial in a primary health care setting in Majorca (Spain). The subjects will be randomised to either Control Group (CG) or Intervention Group (IG). Participants in CG will receive brief advice; participants in IG will receive brief advice with CO-oximetry.

### Study population

The study population is made up of smokers ≥18 years that attend a primary care consultation for any medical problem.

Inclusion criteria will include smokers ≥18 years in the pre-contemplation or contemplation phases described by Prochaska and DiClemente. According to the WHO, a smoker is someone who has smoked daily at least during the past month, irrespective of the amount of cigarettes he/she has smoked. A smoker in the pre-contemplation phase is someone who is not aware that he/she has a problem or thinks he/she is not able to change, does not see his/her behaviour as a reason for concern, and does not consider changes within the next 6 months. A smoker in the contemplation phase is characterised by ambivalence, he/she simultaneously considers and rejects the idea of changing, oscillates between worry and lack of worry as well as between the motivations to change or to continue without changing, and intent to change is established in the long-term without specifying when [9]. Exclusion criteria will include smokers in the preparation phase (prepared to quit smoking in a month’s time); people with a terminal illness or whose health status does not allow them to understand the study or complete the informed consent either due to mental illness or transitory psychiatric deterioration at the moment of inclusion; pregnant or breastfeeding women.

### Recruitment of subjects

Every person consulting a general practitioner (GP) or a nurse will be asked about their smoking behaviour and their attitude toward quitting smoking. To establish the patient’s phase or period of change, he/she will be asked the following: “have you ever seriously thought that you should quit smoking within 1 month?”, following the model of question used in the ISTAPS project [24].

If the subject fulfils the inclusion criteria, information on the study will be released, the subject invited to participate and, upon acceptance, will provide signed informed consent. Subsequently, the participant will be randomised to either CG or IG.

### Sample size

Accepting an alpha risk of 0.05 and a beta risk of 0.20 in a bilateral contrast, and estimating a rate of loss to follow-up of 10%, 471 subjects in both the control group (CG) and the intervention group (IG) are required in order to detect a difference equal or superior to 5% between groups. A proportion of 5% smoking cessation is estimated in the CG. In total, a sample size of 942 subjects is required.

Forty-eight health care professionals from 16 health care centres will participate in the study. It is expected that each professional will include approximately 20 subjects to attain the sample size required.

### Random allocation to study arms

The randomised scheme will be carried out in blocks of 20, using a computer program. The allocation of each patient will be determined using a table of randomised numbers, and this number will be indicated on a report placed in closed, opaque envelopes sequentially numbered. Each professional will have 20 envelopes, one for each subject. The professional will not know the randomisation scheme. The socio-demographic variables, together with those on the smoking habits of the participants that decline to participate in the study, will be collected in a separate report. The inclusion period for subjects will be 12 months.

### Intervention

The intervention applied to the CG will be brief advice, which will be serious, firm, concise, personalised and appropriate for the phase of change. The brief advice attempts to encourage subjects in a clear, firm and personalised way to quit smoking, inform more than oblige, facilitate information on the negative effects of tobacco on health, highlight the main advantages of quitting smoking and ask them to identify and examine the main obstacles when quitting smoking. Once the brief advice to stop smoking has been carried out verbally, the patients will be given a written document with advice and several guidelines. The healthcare professionals will receive training on how to give the brief advice.

In the IG, apart from giving the brief advice in exactly the same way as for the CG, a CO-oximetry test will also be carried out on each subject. Before it is performed, the basics of the test and what it measures will be explained to patients. Once the subject has properly understood how the technique works and if he/she is at an optimum moment for it to be carried out (having not smoked one hour prior to the test/having not smoked for more than 10 hours), the levels of carbon monoxide in expired air will be measured in parts per million (ppm) and recorded in the data collection booklet (DCB) and in a personal report that will be given out to the subject, together with an explanation and definition of CO-oximetry and an interpretation of the possible results.

The two interventions will be carried out by the healthcare professional including the patient in the trial, irrespective of whether he/she is a GP or a nurse. It will only be performed once, during the first visit following the inclusion of the patient into the study. Each professional will conduct one intervention or the other, depending on what intervention is assigned to the subject during randomisation.

The evaluation at months 6 and 12 will be done by a nurse blinded to patient allocation. At 6 months there will be a telephone evaluation and at 12 months an evaluation in the primary health centre.

### Outcome assessment

#### Primary outcome measure

Sustained smoking cessation at 12 months confirmed by a urine cotinine test. It will be considered successful if the values are below 100 ng/ml.

#### Secondary outcome measures

Point smoking cessation at month 6 and/or month 12 confirmed by a urine cotinine test; self-declared smoking cessation at months 6 and 12; reduction in the number of self-declared cigarettes consumed when smoking cessation is not accomplished at months 6 and 12; variation in the phase of process of smoking cessation: from pre-contemplation to contemplation or preparation phase, or from contemplation to preparation phase, at months 6 and 12.

#### Independent measures

Type of intervention: brief advice or brief advice together with CO-oximetry. At baseline, the following information will be obtained: (a) socio-demographic variables: gender, age, social class measured by means of two indicators suggested by the Spanish Society of Epidemiology: educational level and occupation; (b) regarding the patient’s smoking behaviour: number of cigarettes/day and number of years the patient has been smoking (the number of packets/year will be calculated based on this information); concentration of nicotine and tar smoked currently (obtained from the type of tobacco consumed); number of times the patient has tried to quit smoking and reasons for relapse; maximum amount of time without smoking; tobacco consumption during work, with family and/or friends; physical dependency on nicotine using a simplified version of the Fagerström test; phase of the smoking cessation process: Prochaska and DiClemente phases (pre-contemplation, contemplation, preparation, cessation and relapse); any intervention to quit smoking during the past year; (c) other measures: consumption of other legal drugs (alcohol, psychopharmacological drugs); consumption of illegal drugs (cannabis, cocaine and others); basal disease associated with tobacco consumption (Chronic Obstructive Lung Disease, cancer, ischemic myocardiopathy, cerebrovascular disease); other cardiovascular risk factors (HTA, diabetes mellitus, dyslipidaemia, obesity); mental illness.

At months 6 and 12 the following data will be collected: Smoking behaviour/status (does the patient smoke or has he/she quit); date of smoking cessation, whether the patient has quit smoking; time interval without smoking between date of intervention till six month assessment; participation in a programme for smoking cessation since the beginning of the study.

### Study development

*a).* A pilot study will be carried out in two health centres to proceed with the creation of the final DCB based on comments and suggestions from the GP and nurse participants. *b).* Training will be given to the professional participants in the study regarding brief advice and how to carry out the CO-oximetries to ensure that data collection is as homogeneous as possible. The nursing staff contracted for the development of the evaluations will also receive this training. *c).* A total of 3 contacts with each subject will be carried out. The first contact: inclusion-intervention visit will be carried out by the health centre’s professional participants. Two assessment contacts: a 6 month phone assessment and a final assessment at 12 months in the health centre will be performed by the nursing staff contracted for the study who will not have any knowledge of the randomisation scheme. *d).* On the first visit, once the subject has signed the informed consent and been randomised, baseline information will be collected by means of the basal DCB (socio-demographic variables, smoking habits variables, and other variables related to the consumption of drugs, diseases and cardiovascular risk factors). Subsequently, the intervention will be carried out. *e).* Six months after inclusion, the independent and dependent measurements mentioned above will be collected by means of another DCB by phone. *f).* the final assessment will be carried out at 12 months. The DCB will contain the same questions as those administered at 6 months, the urine cotinine test will be used to confirm sustained smoking cessation in those claiming to have quit smoking, and a CO-oximetry will be performed on patients in both groups. g). If a subject requests help to quit smoking during the study, he/she will be aided and referred to his/her GP or nurse to proceed with the intervention. This information will also be recorded.

### Data handling

Every patient included in the study will be assigned a unique identification number created by means of an algorithm based on the health centre, the patient’s initials, and the patient’s date of birth.

The DCBs will be developed using Teleform Desktop v9.1 format, an automated questionnaire reading programme. Teleform contains a verification model that guarantees the quality of data entry. Two security copies of all the data entered will be made every month and stored on a magnetic tape (IBM 3500 backup 400 Gb server).

### Statistical analysis

The statistical analyses will be carried out using the SPSS programme for Windows v 12.

Descriptive, labelled analyses and data refinement: evaluation of the atypical and extreme values (“outliers”), detection and labelling of the lost and/or not applicable values. We will perform descriptive analysis, with continuous variables summarised by their means and standard deviations for normal distributions, and by median and 25th and 75th percentiles for non-normal distributions.

Basal comparative analysis: Comparison of socio-demographic characteristics, smoking behaviour and other variables collected by means of the t Student and chi-squared tests. If normality is not met, non-parametric tests will be applied.

Final comparative analysis: Comparison of the dependent variables in both groups by means of the t Student and the chi-squared tests. If normality is not met, non-parametric tests will be applied. The relevance of the intervention will be determined from the percentage of patients with sustained smoking cessation in both groups - the relative risk (RR), the reduction in relative risk (RRR), the reduction in absolute risk (RAR), and the number of intent to treat patients (ITT) necessary will be calculated. All the analyses will be carried out based on intent to treat. The patients lost to follow-up will be considered as smokers. The level of statistical significance is established at 5% (bilateral).

### Ethical approval

This study protocol was approved by the Mallorca Primary Care Research Committee and Balearic Islands Clinical Research Ethics Committee (IB 985/08 PI).

The healthcare professionals participating in the study will sign a document guaranteeing confidentiality of the data. Before initiating the study, the patients must sign an informed consent, which will be obtained following the recommendations in the Declaration of Helsinki.

The Investigator will ensure that this study is conducted in accordance with the principles of the Declaration of Helsinki, ICH Guidelines for Good Clinical Practice and in full conformity with relevant regulations.

All substantial amendments to the original approved documents will be also sent to an appropriate Ethics Committee and Regulatory Authority for written approval.

The trial staff will ensure that the participants’ anonymity is maintained. All documents will be stored securely and only accessible to trial staff and authorised personnel. The study will comply with the Data Protection Legislation which requires data to be anonymised.

## Discussion

Smoking behaviour is one of the major public health problems. As such, all interventions that can be done to protect health and motivate smoking cessation are welcomed, especially if they are simple, non-aggressive, accessible and feasible [23]. If this intervention is performed in primary care, where 90% of the population come over 5 years, the population effect is highly relevant and of great benefit to Public Health.

Although our intervention is addressed to all current smokers, it has been specially designed for those smokers who, due to their great physical and psychological dependence and their balance between positive and negative smoking cessation factors, opt in favour of smoking maintenance. These smokers require forceful information directly related to the effects on their health to change the balance of these factors and allow an opportunity for smoking cessation to take place.

One of the limitations of studies evaluating personal risk relates to those cases in which the results generated by the technology used are normal and, in consequence, reinforce the smoker’s perception about the harmless effects of smoking. We think that the brief advice can minimize this.

The urine cotinine test could be perceived by patients as another intervention for smoking cessation and, therefore, have an effect on smoking cessation. We consider that carrying out the test on both groups minimises this effect.

## Competing interests

The authors declare that they have no competing interests.

## Authors’ contribution

JR led the design and developed of the study. HG, MR and DM participated in the design and development of the study and critically reviewed the draft. JR, ET and MR will perform the statistical analysis. AP, CA, LG, ME, EG, AU, AB will coordinate the development of the study in their health centres, recruited subjects, performed interventions and critically reviewed the draft. All the authors have read the draft critically, made contributions, and approved the final text.

## Pre-publication history

The pre-publication history for this paper can be accessed here:

http://www.biomedcentral.com/1471-2458/12/322/prepub
